# Interdisciplinary Approaches in Psychiatric Research: From Neural Dynamics to Clinical Applications in Schizophrenia

**DOI:** 10.62641/aep.v53i1.1878

**Published:** 2025-01-05

**Authors:** Francisco José Esteban, Ramón Guevara, Jesús Poza, Sergio Iglesias-Parro

**Affiliations:** ^1^Department of Experimental Biology, University of Jaén, 23071 Jaén, Spain; ^2^Department of Physics and Astronomy, University of Padova, 35122 Padova, Italy; ^3^Department of Signal Theory and Communications and Telematics Engineering, University of Valladolid, 47011 Valladolid, Spain; ^4^Department of Psychology, University of Jaén, 23071 Jaén, Spain

**Keywords:** schizophrenia, interdisciplinarity, brain connectivity, chronnectomics, computational models

## Abstract

This editorial explores the dynamic psychiatric research field by focusing on interdisciplinary approaches to understand the complexity of mental disorders by placing particular emphasis on schizophrenia. It highlights the need to integrate findings from diverse scientific disciplines, such as neuroscience, computational modeling and genomics, to unravel the multifaceted nature of these conditions. The potential of interdisciplinary research to transform our knowledge and the treatment of psychiatric disorders is underscored by moving beyond traditional models and developing more nuanced frameworks to more effectively address these complexities. Thus by combining perspectives from different fields, significant advancements are expected in the diagnosis, treatment and prevention of mental disorders like schizophrenia, and will open new research and clinical practice avenues in psychiatry.

## The Complexity of Psychiatric Disorders

Psychiatric disorders, particularly schizophrenia, present significant challenges to both clinicians and researchers given their complex and multifaceted nature [1]. These conditions involve a wide array of symptoms, including cognitive deficits, mood disturbances and behavioral abnormalities, which often vary vastly between individuals and can fluctuate over time. These disorders’ heterogeneity complicates efforts made to develop a unified understanding of their underlying neural mechanisms. Schizophrenia, for example, encompasses positive symptoms (i.e., hallucinations and delusions), negative symptoms (i.e., social withdrawal and lack of motivation), and cognitive impairments (i.e., impaired attention and working memory), and each one may be driven by different neurobiological processes. As a result, traditional approaches in psychiatry, which tend to focus on static brain imaging, neurochemistry, or symptom-based diagnostics, often fall short explaining this disorder’s full scope.

One of the key issues in treating schizophrenia and other psychiatric conditions is lack of clear, objective biomarkers that can reliably predict disease onset, progression, or response to treatment. This has led to an overreliance on subjective symptom-based diagnosis, which may not fully capture the disorder’s biological diversity. Moreover, many existing treatments for schizophrenia, particularly antipsychotic medications, target dopamine pathways, by addressing only certain symptoms, such as hallucinations and delusions [2]. However, these treatments are often ineffective for the negative and cognitive symptoms that are equally, if not more, disabling for many patients. This has prompted the need for more innovative and integrative approaches that go beyond traditional methods.

## The Potential of Interdisciplinary Approaches

The recent shift in psychiatry toward interdisciplinary approaches reflects acknowledgment that no single perspective can fully account for the complexity of mental disorders. Advances in fields like neuroscience, computational modeling, and genomics are beginning to offer new insights into the multilayered nature of these conditions. For example, neuroimaging techniques like functional magnetic resonance imaging (fMRI), electroencephalography (EEG), and magnetoencephalography (MEG) allow researchers to observe time-varying changes in brain activity, and provide a more dynamic understanding of how different brain regions interact during various cognitive and emotional states [1]. These techniques, combined with computational models that simulate brain network dynamics, can help to identify the brain activity that corresponds to specific symptoms or disease states [3, 4].

Schizophrenia has particularly become a model disorder for exploring these interdisciplinary approaches given its profound impact on multiple domains of brain function, including perception, cognition, and social behavior. Researchers have begun to apply network neuroscience and systems biology approaches to understand how large-scale brain networks, rather than isolated brain regions, contribute to the pathology of schizophrenia [5]. For instance, dysregulation in the connectivity of the default mode network (DMN), which is involved in self-referential thinking, and the salience network, which is responsible for filtering relevant stimuli from the environment, are thought to underlie key symptoms like hallucinations and delusions [6].

Computational models are also playing a crucial role in synthesizing these data by providing a framework for understanding how disruptions in neural connectivity and brain network dynamics can lead to the wide range of symptoms observed in schizophrenia [4]. These models allow researchers to simulate various scenarios and predict how changes in brain activity might be clinically manifested.

## Chronnectomics: Revealing Temporal Dynamics

Chronnectomics is emerging as one of the most promising interdisciplinary fields in psychiatric research for its ability to reveal the temporal dynamics of brain networks in almost real-time [7, 8]. Unlike traditional methods that focus on static representations of brain connectivity, chronnectomic methods examine how these networks fluctuate and reorganize over time [9]. This approach is especially valuable because brain function is inherently dynamic and constantly changes in response to internal states and external stimuli [7]. Traditional static connectivity analyses may overlook these temporal fluctuations and might potentially miss critical information about how brain networks contribute to both normal cognitive processes and the pathology of psychiatric disorders [10, 11]. By focusing on how connectivity patterns shift moment-to-moment, chronnectomics offers a more nuanced understanding of brain function. Hence these patterns allow us to quantify the properties of global network dynamics in interpretable indices, which can shed new light on subtle alterations in the underlying temporal structure of brain activity during both task performance and rest [12].

The relevance of chronnectomics to psychiatric disorders lies in its ability to capture the temporal instability that often characterizes conditions like schizophrenia and depression [7, 13]. Symptoms in these disorders tend to fluctuate, sometimes unpredictably, which may reflect underlying loss of flexibility in the brain’s functional networks. For example in schizophrenia, patients may experience periods of acute psychosis interspersed with periods of relative cognitive stability. All this suggests that the brain’s connectivity patterns are not static, but oscillate between different states [14]. By tracking the brain’s network dynamics over time, chronnectomics can help to identify when and how these fluctuations occur, and can potentially provide new insights into why symptoms are manifested as they are, and may even lead to the characterization of schizophrenia subtypes clustered on the basis of neurophysiological alterations [8, 15, 16].

One of the major advantages of chronnectomics is its potential to identify specific biomarkers associated with the temporal dynamics of brain networks [17]. Biomarkers are crucial in psychiatric research for improving diagnosis, prognosis, and treatment, but the search for reliable and objective markers in disorders like schizophrenia has been challenging [18]. Chronnectomic biomarkers, which reflect the timing and frequency of network fluctuations, could provide a new class of more sensitive diagnostic tools to each patient’s unique neurophysiological patterns. For instance, identifying a network instability pattern that reliably precedes onset of psychotic symptoms in schizophrenia could lead to early interventions that could stabilize these networks before symptoms worsen.

Moreover, chronnectomic biomarkers are promising for developing personalized therapeutic strategies. By mapping an individual’s brain network dynamics, clinicians could tailor interventions to target specific moments of network instability [19]. For example, if a patient with depression exhibits chronnectomic markers of network instability at specific times of the day, treatments could be adjusted to coincide with these times by maximizing their effectiveness.

## Biological Bases of Consciousness

The study of the biological bases of consciousness represents one of the most complex and intriguing challenges in neuroscience. Given its subjective and elusive nature, consciousness has long since fascinated scientists, but its neural mechanisms remain largely unclear [20, 21]. This enigma becomes particularly relevant in the psychiatric disorders context, where disruptions in consciousness can be both profound and revealing. Schizophrenia particularly offers a unique framework for exploring altered states of consciousness because it is characterized by significant distortions in perception, cognition and behavior. As previously mentioned, patients with schizophrenia often experience hallucinations, delusions and disorganized thinking, and these symptoms reflect dramatic changes in how they perceive and interact with the world around them. These phenomena suggest that the mechanisms underlying consciousness may be disrupted in this disorder [22].

Schizophrenia’s unique symptomatology, such as auditory and visual hallucinations, offers critical insights into the neural circuits responsible for sensory processing and the brain’s ability to differentiate between internal thoughts and external reality. The vivid and often overwhelming nature of these experiences highlights a breakdown in the brain’s ability to maintain normal boundaries between what is real and what is imagined [23]. This breakdown suggests that consciousness relies heavily on precise coordination between the brain regions responsible for perception, attention and memory. In schizophrenia, disruptions in this coordination may lead to the fragmented experiences that characterize the disorder, which could offer valuable clues about the role that neural networks play in sustaining a coherent sense of self and reality.

Recent advances in neuroimaging and electrophysiology have begun to reveal specific patterns of the brain network dysfunction that is associated with altered states of consciousness in schizophrenia [13]. For instance, research has shown that patients with schizophrenia often exhibit hyperconnectivity within the DMN, a brain network implicated in self-referential thinking and mind-wandering, and disconnectivity between the DMN and other networks that are responsible for processing external stimuli [11]. This imbalance may help to explain why individuals with schizophrenia struggle to differentiate between internal thoughts and external events, which leads to hallucinations and delusions.

Moreover, temporal brain activity dynamics, as revealed by electrophysiological techniques, have provided new insights into how consciousness is disrupted in schizophrenia [24]. Abnormalities in oscillatory activity, particularly within the gamma frequency range, have been linked with altered conscious perception [25]. Gamma oscillations are thought to play a crucial role in synchronizing activity across different brain regions by enabling the integration of sensory information into a cohesive conscious experience [26]. In schizophrenia, disturbances in gamma oscillations may contribute to the fragmented and disordered thought patterns that are hallmark symptoms of the disease [27]. The potential clinical applications of this research are significant. By mapping the neural correlates of consciousness in schizophrenia, scientists can begin to identify biomarkers of altered consciousness that can be used to more effectively diagnose and monitor the disorder [28]. Additionally, these findings open the door to developing therapeutic interventions that directly target the neural dynamics of consciousness [8]. For example, neuromodulation techniques such as transcranial magnetic stimulation (TMS) and transcranial direct current stimulation (tDCS) are being explored as potential treatments for modulating dysfunctional brain networks in psychiatric conditions [29]. Moreover, the informational structures [30]—topological representations of brain activity at each moment—have enabled the classification of distinct states of consciousness by leveraging the time-dependent statistics of these structures, which have been previously concealed within neuroimaging dynamics, to achieve high precision in distinguishing resting-state Blood-Oxygen-Level-Dependent fMRI signals from patients in various conditions [26].

## Mathematical Models: Simulating Brain Complexity

Mathematical models have emerged as a crucial tool in the effort to understand the intricate and often unpredictable nature of psychiatric disorders. Disorders like schizophrenia, characterized by chaotic brain activity, pose significant challenges for both diagnosis and treatment [31]. Traditional approaches often struggle to capture the disorder’s full scope given its multifaceted symptoms. These symptoms are thought to arise from disruptions in large-scale neural networks. However, the exact mechanisms that lead to such disruptions remain elusive. This is where mathematical models become invaluable because they offer a structured, quantitative approach to analyze the complex and nonlinear brain dynamics that underlie psychiatric conditions [32].

Abnormal brain activity patterns, which are extremely variable over time and difficult to predict, lie at the core of schizophrenia and many other psychiatric disorders [27]. Brain regions that normally communicate in a synchronized manner may become desynchronized, which leads to disturbances in cognition, perception and behavior [25]; early on this led to schizophrenia being identified as a “disconnection syndrome” [33]. For example, aberrant connectivity between the prefrontal cortex and the hippocampus is thought to contribute to the cognitive deficits seen in schizophrenia, while overactivity in the dopaminergic system is linked with the disorder’s positive symptoms, such as hallucinations [34]. Mathematical models allow researchers to simulate these disordered interactions in a controlled environment and offer a way to disentangle the complex network of interactions that contribute to the disorder [35].

One of the strengths of mathematical modeling in psychiatry is its ability to distill large amounts of complex data into simplified, yet informative, frameworks [36]. By representing the brain as a network of interconnected nodes, mathematical models can help to identify which regions are more affected by the disorder, how information flow is disrupted, and how these disruptions correlate with specific clinical symptoms. For instance, graph theory-based models can reveal changes in network topology, such as a shift from a more organized, small-world network to a more random, chaotic one, which is often seen in schizophrenia [37, 38]. These models provide insights into how the brain’s overall architecture is altered in psychiatric conditions, which leads to new hypotheses about the disorder’s etiology and progression.

Apart from shedding light on psychiatric disorders’ underlying mechanisms, mathematical models open up new possibilities for developing more targeted and effective interventions [39]. As these models can simulate the effects of different interventions on brain network dynamics, they offer a powerful tool for testing potential treatments before they are applied in clinical settings. For example, by modeling the effects of neuromodulation techniques, such as transcranial TMS or deep brain stimulation (DBS), on disrupted neural circuits, researchers can predict how these treatments might restore normal brain function [40].

## Translating Research into Clinical Practice

The final and arguably most critical step in the interdisciplinary effort to advance psychiatric research lies in the translation of scientific findings into clinical practice. While advances in neuroscience, computational modeling, and chronnectomics have provided groundbreaking insights into the underlying mechanisms of psychiatric disorders, the challenge remains in applying these insights to the treatment of real patients. Schizophrenia, with its heterogeneous presentation, vividly illustrates the complexity of this translational process. The disorder is manifested within a wide range of symptoms. This variability in symptoms from patient to patient, and even within the same individual over time, makes it particularly difficult to design universally effective treatment strategies.

As discussed earlier, interdisciplinary research that combines neuroscience, computational modeling and genomics is essential for understanding psychiatric disorders. One of the key opportunities for improving patient care lies in the incorporation of neurobiological data, such as structural and functional brain imaging, into clinical decision making [35]. Neuroimaging can reveal abnormalities in brain connectivity and activity patterns that are associated with specific symptoms or disease subtypes [15].

In addition to neuroimaging data, chronnectomic findings—which reveal how brain networks fluctuate over time—offer a dynamic perspective on schizophrenia’s pathology [9]. Chronnectomics helps to capture the temporal instability of brain networks that often correlates with symptom variability. For example, fluctuations in network connectivity may be associated with episodes of acute psychosis or cognitive decline in schizophrenia patients. By mapping these fluctuations, researchers can identify critical windows in which therapeutic interventions might be more effective by allowing for more proactive and timely treatment. This temporal understanding of brain dynamics represents a significant shift from static brain models for offering a more personalized approach to understanding how schizophrenia is manifested in individual patients.

Computational models also play a pivotal role in bridging the gap between scientific research and clinical practice [31]. These models can simulate brain activity and predict how alterations in neural connectivity impact behavior and cognition. In the clinical setting, computational models can be used to create individualized maps of a patient’s brain dynamics by highlighting specific regions or networks that are disrupted [32]. This level of granularity enables clinicians to design treatment plans that are tailored to each patient’s neurodynamic profile, a central concept to precision medicine. For instance, if a computational model indicates that a patient’s hallucinations are linked with hyperconnectivity in the brain’s auditory processing regions, treatments can be specifically targeted to modulate activity in those areas.

The integration of these data sources (neuroimaging, chronnectomics and computational modeling) into clinical practice offers a powerful means to personalize treatment strategies in psychiatry [39]. Rather than relying on a one-size-fits-all approach, clinicians can use this interdisciplinary knowledge to tailor interventions based on each patient’s unique neurobiological and neurodynamic profile. This can involve fine-tuning pharmacological treatments, adjusting the timing and intensity of cognitive behavioral therapies or implementing neuromodulation techniques, such as TMS or tDCS at specific time points when the brain is more susceptible to change.

This bridging of basic science and clinical practice is not only essential for personalizing treatment but also for advancing therapeutic innovations in psychiatry [41]. By continuously integrating new research findings into clinical workflows, psychiatric care can evolve in a more informed and data-driven direction. For instance, as new biomarkers are identified through chronnectomic studies, they can be incorporated into diagnostic tools that provide more accurate and earlier identification of psychiatric disorders [42], potentially before the most severe symptoms arise. Similarly, computational models can help clinicians predict how a patient might respond to a particular treatment [4], which would allow for more effective interventions and personalized treatment strategies that address individual patients’ unique neurodynamic profiles.

Ultimately, this interdisciplinary approach, which brings together neurobiology, a dynamic brain network analysis and computational modeling, has immense potential to transform the psychiatry field; the combination of the research findings from these different knowledge areas can be essential for identifying the biological substrates of a heterogeneous syndrome like schizophrenia. By advancing therapeutic innovations and improving patient outcomes [35], the insights gained from cutting-edge research are translated into tangible benefits for individuals affected by complex psychiatric disorders like schizophrenia. With this integration of science and clinical care [41], psychiatry can move toward a future in which treatments are more precise, personalized and effective.

## Conclusion

Taken together, the convergence of neuroscience, chronnectomics, mathematical modeling and clinical research is transforming the understanding of psychiatric disorders, particularly schizophrenia. By embracing an interdisciplinary approach, researchers and clinicians are moving beyond traditional models and developing more nuanced frameworks to understand and treat these complex conditions, whose ultimate aim is to improve the lives of individuals affected by psychiatric disorders.

## Availability of Data and Materials

Not applicable.
